# GABA promotes gastrin-releasing peptide secretion in NE/NE-like cells: Contribution to prostate cancer progression

**DOI:** 10.1038/s41598-018-28538-z

**Published:** 2018-07-06

**Authors:** Susana R. Solorzano, Ivan Imaz-Rosshandler, Ignacio Camacho-Arroyo, Pilar García-Tobilla, Gustavo Morales-Montor, Patricia Salazar, Ma. Leticia Arena-Ortiz, Mauricio Rodríguez-Dorantes

**Affiliations:** 1 Oncogenomics Laboratory, Instituto Nacional de Medicina Genómica, Mexico City, 14610 Mexico; 2 DAMTP, Centre for Mathematical Sciences, University of Cambridge, Cambridge, CB3 OWA UK; 3 Unidad de Investigación en Reproducción Humana, Instituto Nacional de Perinatología-Facultad de Química, Universidad Nacional Autónoma de México (UNAM), Mexico City, Mexico; 4 Urology Department, Hospital General Dr. Manuel Gea González, Mexico City, 14080 Mexico; 5 Department of Neurosciences, Instituto de Fisiología Celular, UNAM, AP 70/253, 04510, Mexico City, Mexico; 6 Ecogenomics Studies Laboratory PCT, Facultad de Ciencias, UNAM, Mérida, Yucatán, Mexico

## Abstract

In prostate cancer (PCa), neuroendocrine cells (NE) have been associated with the progression of the disease due to the secretion of neuropeptides that are capable of diffusing and influence surrounding cells. The GABAergic system is enriched in NE-like cells, and contributes to PCa progression. Additionally, γ-aminobutyric acid (GABA) stimulates the secretion of gastrin-releasing peptide (GRP) in peripheral organs. For the first time, in this study we show the role of GABA and GABA_B_ receptor 1 (GABBR1) expression in GRP secretion in NE-like prostate cancer cells. We demonstrated an increase in GRP levels in NE-like cell medium treated with GABA_B_ receptor agonist. Moreover, the blocking of this receptor inhibited GABA-induced GRP secretion. The invasive potential of PC3 cells was enhanced by either GRP or conditioned medium of NE-like cells treated with GABA. Additionally, we confirmed a positive correlation between GABA and GRP levels in the serum of PCa patients with NE markers. Finally, using public available data sets, we found a negative correlation between *GABBR1* and androgen receptor (AR) expression, as well as a strong positive correlation between *GABBR1* and enolase 2. These results suggest that GABA via GABBR1 induces GRP secretion in NE like cells involved in PCa progression.

## Introduction

Despite great efforts to improve treatment, prostate cancer (PCa) is the most frequently diagnosed cancer among men in developed countries^1^. Androgen ablation has been the main therapeutic intervention in managing hormone-sensitive prostate cancer^2^. However, in most cases, tumors tend to progress, despite treatment, to the castration-resistant prostate cancer (CRPC) stage. Once this occurs, the median survival rate is 18 to 24 months^3^. CRPC is a lethal stage, when prostate cancer progresses and metastasizes^4^.

Hormone-treated and hormone-refractory tumors can undergo neuroendocrine differentiation (NED)^5,6^. NED is characterized by an increase in a malignant subpopulation of cells with neuroendocrine (NE) features. Among CRPC tumors, it is estimated that 40–100% acquires NED. These tumors are referred to as neuroendocrine prostate cancer (NEPC)^7,8^. Experiments conducted *in vitro* and *in vivo* (animal models) have shown that prostate adenocarcinoma cells can transdifferentiate and acquire a NE phenotype through a process termed NE transdifferentiation^9–12^. These cells are typically referred to as “NE-like” cells, because their origin and biochemical characteristics are different from those of normal NE cells^9,10^. Wright *et al*. demonstrated that a knockdown of the androgen receptor (*AR*) in LNCaP cells induced a NE-like morphology and expression of NE markers. Those findings suggested that AR activity repressed the transdifferentiation process^11^.

NE transdifferentiation in PCa revealed an epithelial plasticity that gave rise to tumor adaptation in response to AR-targeted therapies. A recent study showed that NEPC occurred in 30/81 patients with CRPC. Moreover, *AR* expression was low in these tumors^12^. Although NE cells and NE-like cells do not express AR^13^, they can secrete many types of neuropeptides, like gastrin-releasing peptide (GRP), serotonin, and neurotensin, and they express NE markers, such as enolase 2 (ENO2), chromogranin A, and chromogranin B^6,14^. Hence, understanding the molecular etiology of NEPC and identifying novel therapeutic targets are of utmost importance, particularly because, at present, no effective therapy is available.

Neuropeptides, such as GRP, have been positively associated with PCa progression^15^. GRP, a 27-amino acid neuropeptide, is the mammalian homologue of the peptide known as bombesin, which was isolated from frog skin^16^. Neuroendocrine cells in tumors are considered the main source of GRP. In PCa, GRP stimulates mitogen-stimulated proliferation, migration, and invasion, through autocrine and paracrine signaling^17,18^. Studies have shown that serum GRP concentrations were elevated in patients with advanced PCa. Specifically, GRP concentrations were significantly elevated in advanced metastatic and CRPC tumors, but not in the early cancer stages^19^.

The Gordon group created transgenic mice (CR2-TAg) that developed a pattern of NEPC initiation and progression. Prostate samples from CR2-Tag mice were compared to prostate samples from normal mice with GeneChip arrays to identify candidate mediators of NE cell differentiation. One candidate gene was glutamic acid decarboxylase (Gad1 in mouse, GAD1 in human), which showed 40-fold higher expression in NEPC than in normal NE cells^20,21^. The GAD1 enzyme produces the most abundant inhibitory neurotransmitter in the mammalian brain: γ-aminobutyric acid (GABA). GABA plays a tissue-specific function^22–28^, and it is widespread throughout periphery organs, including the prostate.

The above-mentioned study also showed that GABA levels were enriched in NE-like cells, compared to normal NE cells. Likewise, NE-like cells expressed functional GABA_B_ receptors (GABBR1), which regulated the invasion of PCa cells and promoted matrix metalloproteinase (MMP) expression^29–31^. GABBR1 is a metabotropic G-protein-coupled receptor that mediates the inhibitory effects of GABA; these effects play crucial roles in hepatocellular and pancreatic carcinomas^32^. However, how GABA participates in the invasion of PCa cells remains unknown.

GABA has been reported to regulate the release of neuropeptides and hormones in different peripheral organs. For instance, it governs somatostatin and glucagon secretion in pancreatic beta cells^33^; and in the stomach, it regulates the secretion of gastrin, somatostatin, and GRP by endocrine cells^34^. In this study, we investigated the role of GABA in GRP secretion in NE/NE-like cells derived from PCa samples, and its impact in PCa progression. We demonstrated that GABA, through GABBR1, induced GRP secretion. The results suggested that GABA might be a central player in regulating PCa progression when tumors lack the AR.

## Results

### Establishment of a NE-like cell line with siRNA-mediated AR silencing in LNCaP cells

**Figure 1:**
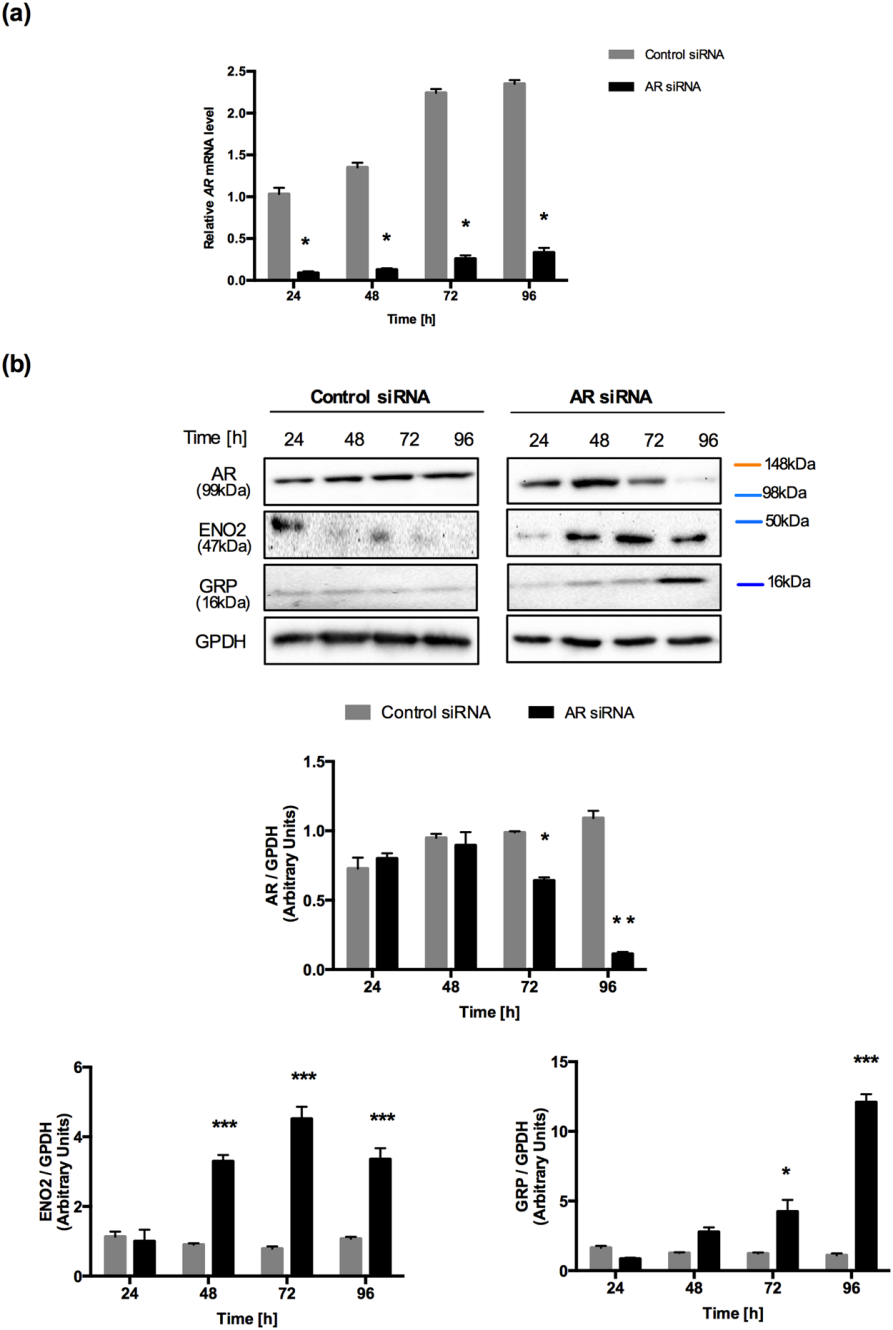
Establishment of NE-like cell line by siRNA targeted to the AR silence in LNCaP cell. (**a**) *AR* mRNA expression levels were analyzed by RT-qPCR in LNCaP cells transfected with AR-specific siRNA and control siRNA from 24 h to 96 h in RPMI supplemented with 10% FBS. Experiments were repeated four times. Differences among groups were compared by two-tailed unpaired Student’s t test, *p < 0.0001. (**b**) Upper panel, Representative Western blot analysis of AR, ENO2 and GRP expression in LNCaP cells transfected with AR-specific siRNA and Control siRNA from 24 h to 96 h. GPDH was used as a loading control. Lower panel, ImageJ software was used for densitometric quantification of proteins. Graphs represent the mean ± SEM (n = 3) *p < 0.05, **p < 0.01 and ***p < 0.001 compared to the control group. The samples derive from the same experiment and that gels/blots were processed in parallel. Full-length blots/gels are presented in Supplementary Fig. 1.

Wright *et al*. reported that an *AR* knockdown induced neuronal-specific expression of proteins, like ENO2, which led to a NE phenotype in LNCaP cells^11^. We decided to use *AR*-specific siRNA in LNCaP cells to establish a NE cell model. LNCaP cells transfected with an *AR*-specific siRNA exhibited successful *AR* mRNA knockdown at 24 h to 96 h (>70% knockdown) compared to cells transfected with control siRNA (*p < 0.0001; Fig. 1a). At the protein level, AR could not be detected at 96 h (**p < 0.01); in contrast, AR protein levels remained unchanged in control cells (Fig. 1b). Furthermore, we confirmed that the *AR* knockdown led to an elevation in the ENO2 content (***p < 0.001). GRP was also evaluated as a NE protein, as previously described^35^. We observed that GRP protein levels were also elevated in *AR*-silenced cells (*p < 0.05 and ***p < 0.001) (Fig. 1b). These results confirmed the hypothesis of a potential link between AR inactivation and the increased frequency of NE cells in androgen-independent tumors^11^.

### Presence of GABA in LNCaP cells after knocking down AR with siRNA

**Figure 2:**
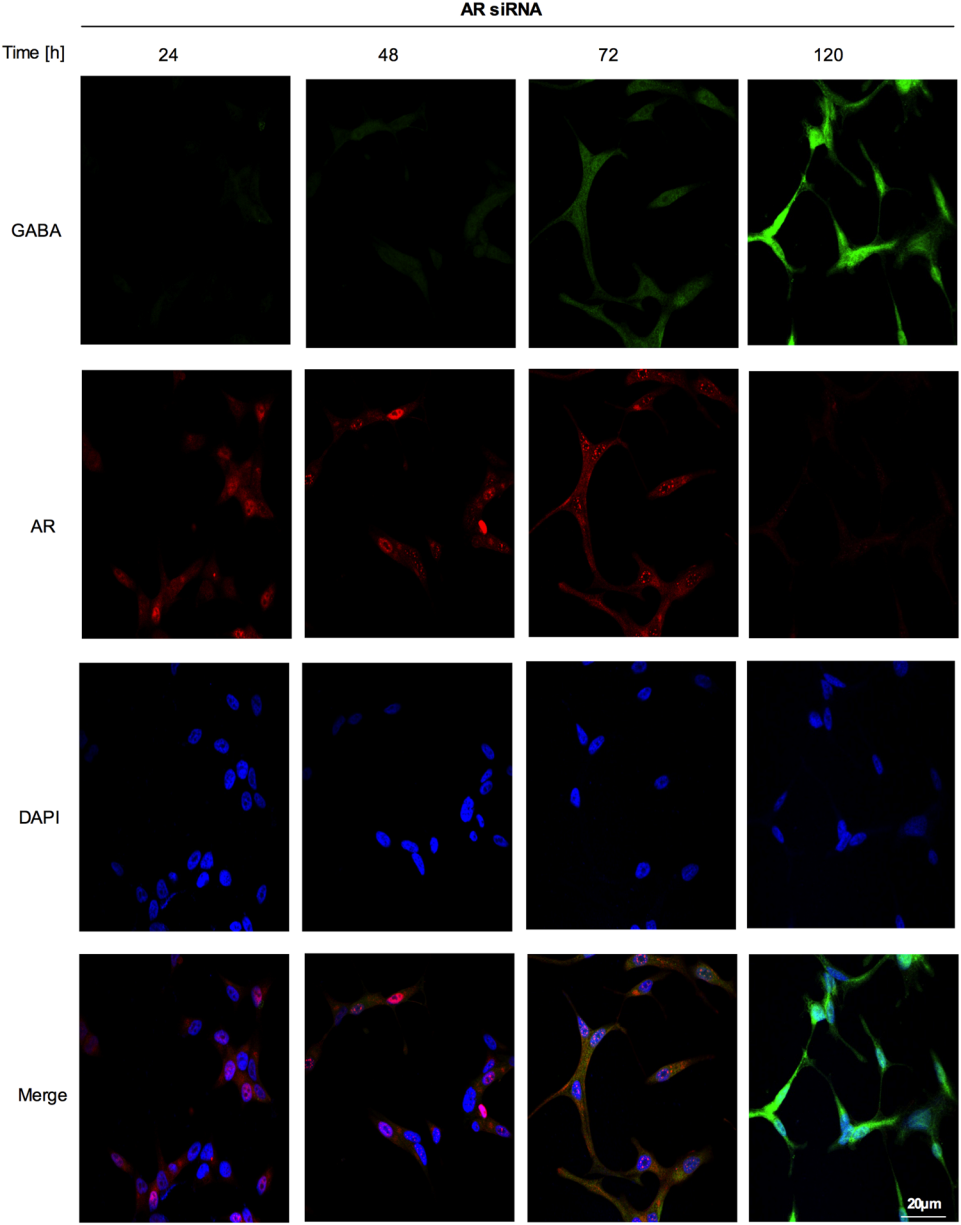
GABA expression is induced in NE-like cells. Fluorescence immunohistochemistry was performed to examine GABA and AR content in LNCaP cells transfected with AR-specific siRNA from 24 to 96 h. An anti-GABA antibody (green staining), anti-AR antibody (red staining) and 4,6-diamidino-2-phenylindole (DAPI) were used. GABA levels were increased after AR silencing. Morphological changes can be seen as well. The proportion of LNCaP cells with obvious protrusions showed an increase once GABA levels were increased and AR was silenced.

It has been reported that GABA and its receptor were present at higher levels in NEPC cells, compared to other prostate cell types^30^. This observation prompted to examine whether GABA was present in LNCaP cells after the *AR* knockdown. Thus, at 96 h, we found that AR levels decreased and GABA levels increased. We also observed that the *AR* knockdown induced morphological changes in LNCaP cells. At 96 h, cells showed increases in protrusions and extended processes (Fig. 2). Control siRNA did not lead to any morphology changes or changes in GABA levels (Supplementary Fig. 2). Taken together, our results suggested that the acquisition of the NE phenotype was linked to an increase in GABA levels and a decrease in AR expression in LNCaP cells.

### Secretion of GABA vesicles from NE-like cells mediated by GABBR1

**Figure 3:**
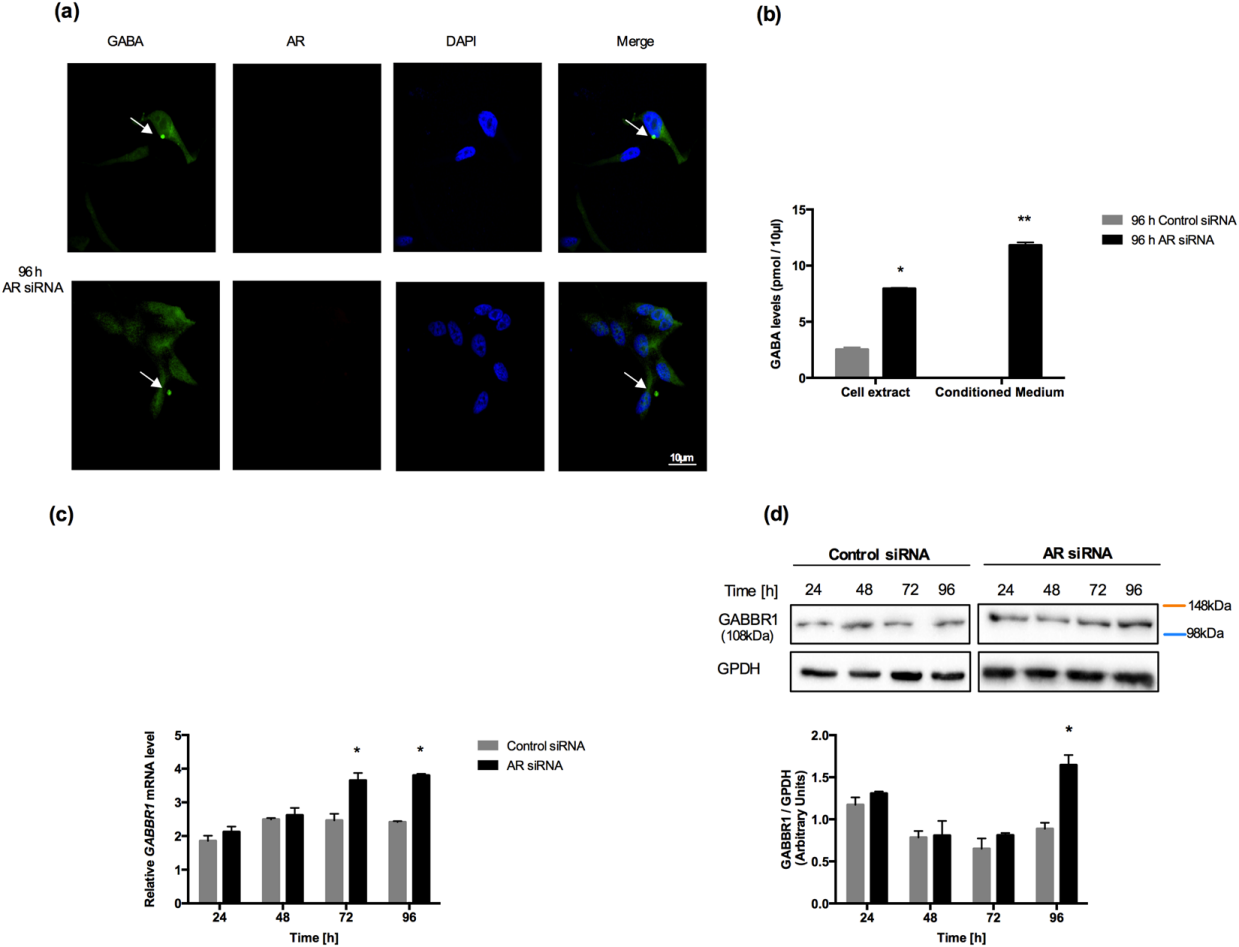
Secretion of GABA vesicles in NE-like cells mediated by GABBR1. (**a**) Representative immunofluorescence detection of GABA vesicles (green staining) at 96 h in AR siRNA LNCaP cells. The presence of these vesicles was associated with the reduction in AR expression and the display of the neuroendocrine phenotype. Vesicles are indicated by arrows; anti-AR antibody (red staining) and 4,6-diamidino-2-phenylindole (DAPI) were used; (**b**) GABA levels were evaluated by HPLC in 96 h LNCaP cells transfected with AR-specific siRNA and control siRNA from an aliquot of total lysate proteins and conditioned medium. Experiments were repeated three times. Differences among groups were compared by two-tailed unpaired Student’s t test, *p < 0.01 and **p < 0.001; (**c**) *GABBR1* mRNA expression levels were examined by RT-qPCR in LNCaP cells transfected with AR-specific siRNA and control siRNA. Experiments were repeated three times. Differences among groups were compared by two-tailed unpaired Student’s t test, *p < 0.0001. (**d**) Western blot analysis of GABBR1 after transfection with AR-specific siRNA and control siRNA from 24 h to 96 h. GPDH was used as a loading control; representative blots are shown of three replicated experiments (Upper panel). Intensity of the bands was quantified with ImageJ software and GABBR1/GPDH is shown in the bar graph (Lower panel) *p < 0.05. The samples derive from the same experiment and that gels/blots were processed in parallel. Full-length blots/gels are presented in Supplementary Fig. 1.

Interestingly, we found that LNCaP cells were able to secrete GABA vesicles 96 hours after the *AR*-knockdown (Fig. 3a, Supplementary Fig. 3). To evaluate further whether NE-like cells secreted GABA, we used HPLC to measure GABA levels in the cell extracts and conditioned medium of *AR*-knockdown and control cells at 96 h. We found increased GABA levels in cell extracts of *AR*-knockdown LNCaP cells (*p < 0.01; Fig. 3b), but not in the conditioned medium of cells transfected with control siRNA (**p < 0.001; Fig. 3b).

We then assessed the expression of GABA_B_ receptors, based on evidence from previous reports, which suggested that these receptors were implicated in promoting metastasis, particularly in NEPC tumors^12,36^. *GABBR1* expression was induced by ~1.2-fold at 72 h after *AR*-knockdown in LNCaP cells. This expression level was maintained until 96 h after silencing (*p < 0.0001; Fig. 3c). In contrast, *GABBR2* mRNA levels did not change with the *AR*-knockdown in LNCaP cells (Supplementary Fig. 4). The GABBR1 protein content increased by ~0.8-fold at 96 h (*p < 0.05) (Fig. 3d), when AR was undetectable. Thus, we showed that both GABA levels and GABABR1 expression were elevated in NE-like cells.

### Regulation of GRP by exogenous and endogenous GABA-GABBR1 in NE-like cells

**Figure 4:**
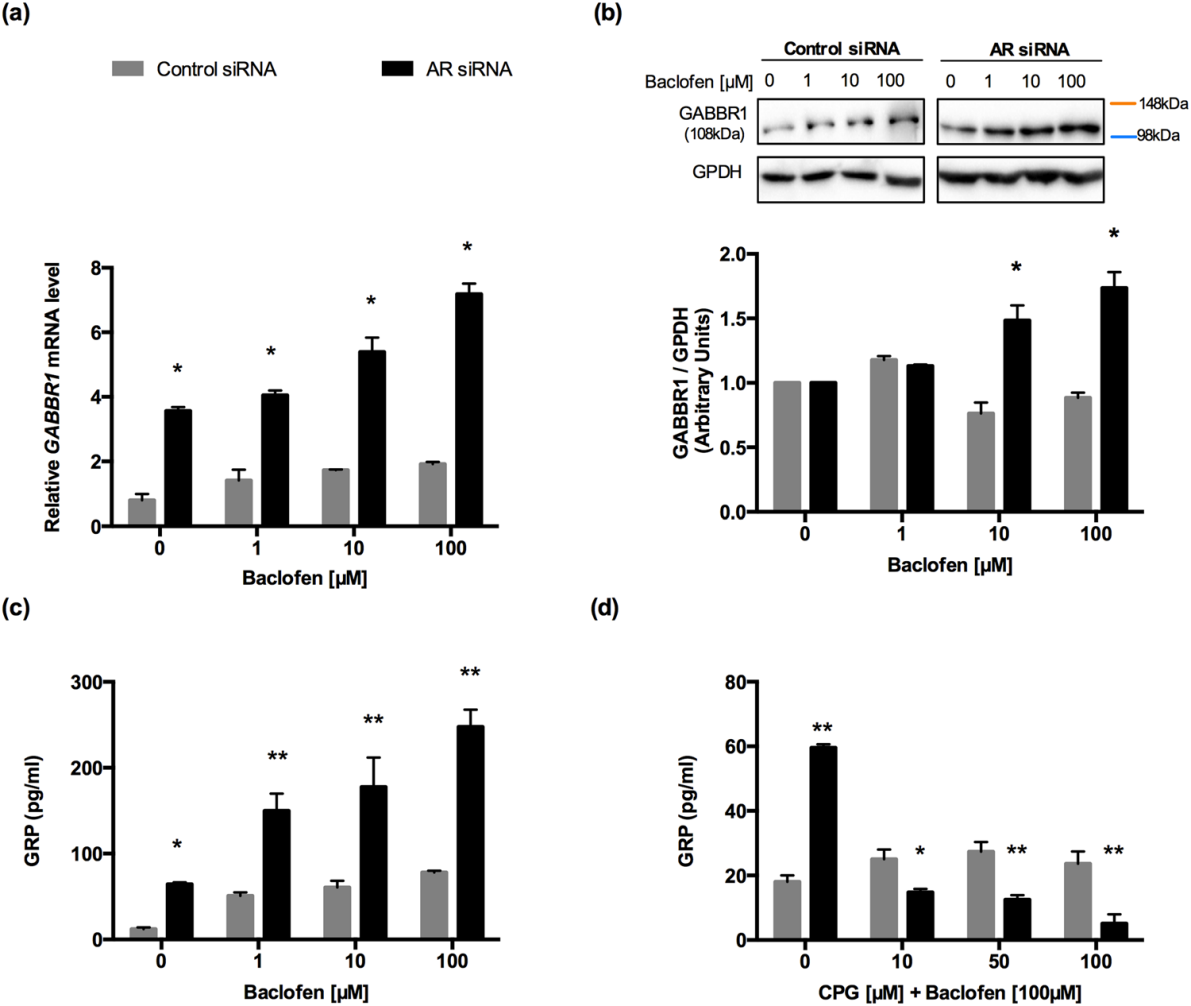
GABA promotes GRP secretion mediated by GABBR1 in NE-like cells. (**a**) *GABBR1* mRNA expression was evaluated by RT-qPCR of 96 h AR siRNA cells and 96 h control siRNA cells grown for 12 h in RPMI with indicated concentrations of baclofen. Differences among groups were compared by two-tailed unpaired Student’s t test, *p < 0.0001. (**b**) GABBR1 protein expression was evaluated by Western Blot in whole cell lysate. GAPDH was used as a loading control. Graphs represent the mean ± SEM (n = 4), *p < 0.001. The last result was normalized to 1 with respect to the vehicle. The concentrations of GRP in the medium of 96 h AR siRNA cells and 96 h control siRNA cells were examined by ELISA (n = 3/group). (**c**) GRP was present in the medium of both cells incubated with RPMI containing different concentrations of Baclofen (GABA_B_ agonist) 1, 10 and 100 µM for 12 h. Differences among groups were compared by two-tailed unpaired Student’s t test, *p < 0.001 and **p < 0.0001. (**d**) GABA_B_ antagonist (CGP 35348) was used, and cells were treated with 10, 50 and 100 µM in combination with baclofen (100 µm) for 12 h. Differences among groups were compared by two-tailed unpaired Student’s t test, *p < 0.001 and **p < 0.0001. The samples derive from the same experiment and that gels/blots were processed in parallel. Full-length blots/gels are presented in Supplementary Fig. 1.

We explored whether GABBR1 participated in the secretion of GRP (involved in cell invasion), as described previously in studies on stomach endocrine cells^34,36,37^. We studied the expression of GABBR1 in NE-like and control cells treated with baclofen. The expression of *GABBR1* in NE-like cells increased with increases in baclofen concentrations (*p < 0.0001; Fig. 4a). In a Western blot analysis, the GABBR1 content was elevated in NE-like cells treated with 10 and 100 µM of baclofen (*p < 0.001; Fig. 4b). Moreover, the GRP concentration was higher in the medium of NE-like cells treated with baclofen compared to that of control cells (Fig. 4c). To confirm the role of GABA_B_ receptor in GRP secretion, we exposed NE-like cells and control cells to the GABA_B_ receptor antagonist, CPG 35348 (10, 50, and 100 µM), for 12 h, combined with baclofen (100 µM). We observed that CPG 35348 blocked the baclofen effects on GRP secretion. In fact, at the highest concentration, CPG 35348 inhibited GRP secretion (Fig. 4d). These results suggested that GABBR1 activation stimulated GRP secretion in NE-like cells.

### Modification in the invasive capacity of PC3 cells by conditioned medium from NE-like cells

**Figure 5:**
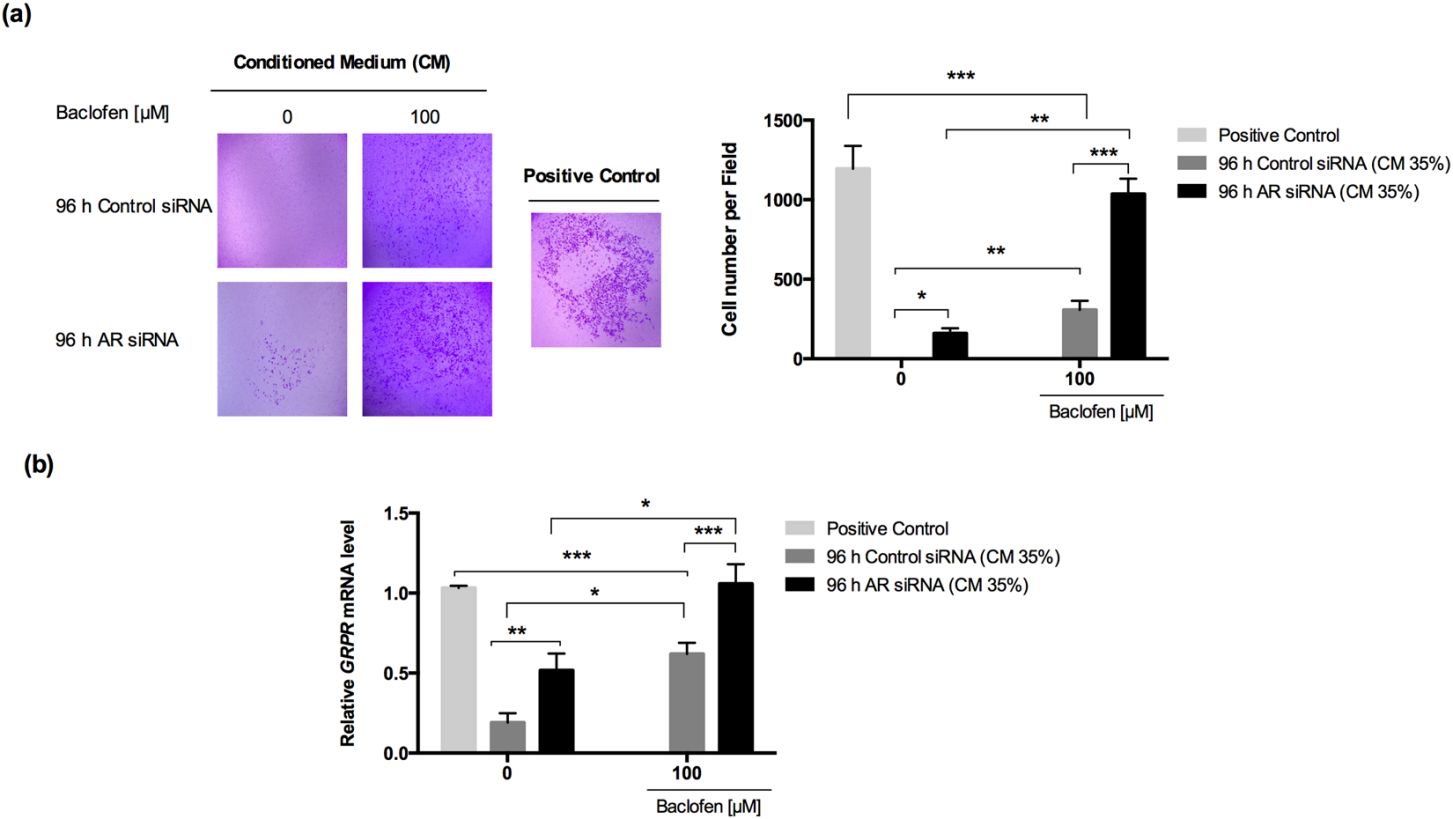
The conditioned medium of NE-like cells modifies the invasive capacity of PC3 cells through GRPR. (**a**) Invasion assay was performed by using a Boyden chamber. PC3 cells (6 × 10^4^) suspended in 200 µl of culture medium without FBS were placed in the upper chamber. 700 µl of conditioned medium from 96 h Control siRNA and 96 h AR siRNA treated with or without baclofen (100 µM) was added in the lower chamber for 6 h. Results are represented as mean ± SEM of three independent experiments in triplicate wells, *p < 0.05, **p < 0.001 and ***p < 0.0001. (**b**) *GRPR* mRNA expression was analyzed by RT-qPCR. Results are presented as mean ± SEM of three independent experiments in triplicate, *p < 0.05 **p < 0.001 and ***p < 0.0001. GRP synthetized was used as positive control; the previous concentration represents 35% of conditioned medium from 96 h AR siRNA treated with baclofen (100 µM) that was used to perform the experiments.

We collected conditioned media from NE-like and control cells treated with or without 100 µM of baclofen for 12 h. With a Boyden chamber assay, we evaluated whether the conditioned media could modify the invasive capacity of PC3 cells. We applied synthesized GRP as a positive control for inducing invasion. The upper chamber contained PC3 cells (6 × 10^4^) suspended in 200 µl of culture medium. The lower chamber contained 700 µl of either conditioned medium or GRP (positive control). Samples were incubated for 6 h, as described previously^38^. We found that PC3 cell invasion was significantly augmented by the conditioned medium from NE-like cells (Fig. 5a). Interestingly, incubation in the conditioned medium from NE-like cells without baclofen treatment significantly increased PC3 cell invasion compared to conditioned medium from control cells (Fig. 5a).

In addition, we incubated PC3 cells with 35% of conditioned medium from either NE-like cells or control cells, treated with and without baclofen (100 µM) for 24 h. We performed RT-qPCR on cell extracts, and found a significant increase in *GRPR* expression in PC3 cells treated with conditioned medium from baclofen-treated NE-like cells (Fig. 5b). These results suggested that GRP secreted by NE-like cells increased PC3 invasion by activating *GRPR* in AR-deficient cells. Similar results were observed in *AR*-knockdown LNCaP cells at 96 h, when they were stimulated with the conditioned medium described above (Supplementary Fig. 5).

### Positive correlations between GABA and GRP levels in patients with NE characteristics

**Figure 6:**
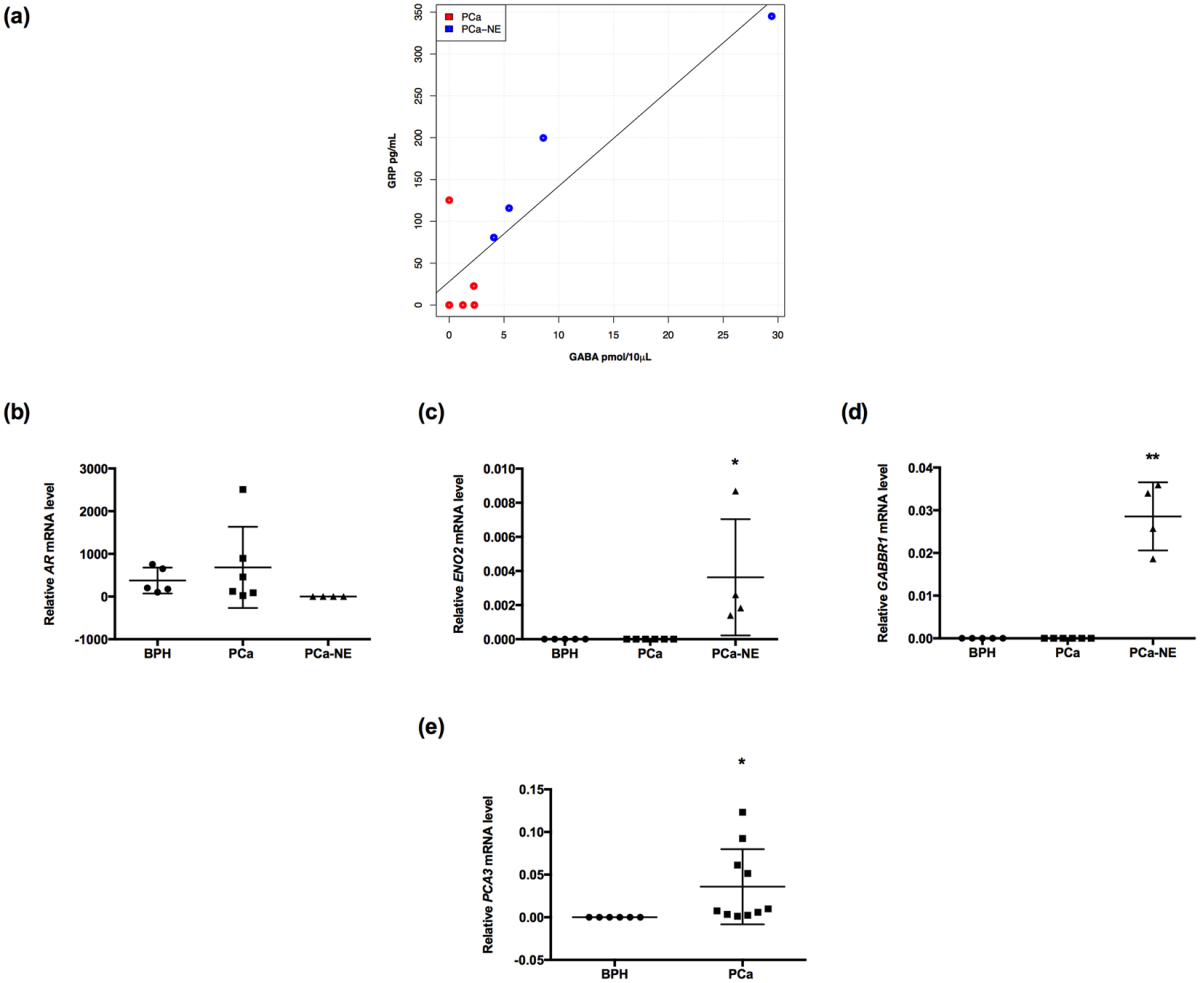
GABA levels are positively correlated with GRP levels in patients with NE markers. (**a**) A positive correlation between GABA and GRP levels was found in PCa patients (r = 0.894; p = 0004; n = 10), and when patients were separated, PCa-NE patients had a strong positive correlation (r = 0.961; p = 0.03; n = 4). (**b**–**d**) Relative *AR*, *ENO2* (*p < 0.05), *GABBR1* (**p < 0.001) gene expression in BPH vs. PCa and BPH vs. PCa-NE, respectively. *AR* mRNA levels were not significantly different between groups. (**e**) *PCA3* mRNA levels were significantly higher in PCa patients compared to BPH patients (*p < 0.05).

To investigate whether GABA and GRP levels were correlated, we evaluated serum samples from ten patients with PCa. We observed a positive correlation between GABA and GRP levels in all samples (correlation coefficient [r] = 0.894; p = 0.0004). The mean ± SEM serum concentrations were: GRP, 88.92 ± 35.91 pg/ml; and GABA, 5.33 ± 2.8 pmol/10 µl. When the samples were separated into two groups according to their characteristics, the group with NE characteristics showed the strongest positive correlation (r = 0.961; p = 0.03; Fig. 6a).

We also measured levels of *AR*, *ENO2*, and *GABBR1* in urine samples from patients with PCa (n = 10) and patients with Benign Prostatic Hyperplasia (BPH) (n = 5). The NEPC group displayed the lowest *AR* levels, compared to the other two groups (PCa and BPH) (Fig. 6b). The NEPC group also displayed elevated *ENO2* expression (*p < 0.05; Fig. 6c) and elevated *GABBR1* mRNA levels (**p < 0.001; Fig. 6d). As a control for the diagnosis of PCA, *PCA3* expression was assessed with RT-qPCR in urine samples from patients with BPH and PCa (*p < 0.05; Fig. 6e)^39^. Keeping in mind the limited number of samples, these results pointed to a strong correlation between GABA and GRP in patients with NE characteristics.

### Correlations between *GABBR1* and *AR* and *GABBR1* and *ENO2* expression in NEPCs

**Figure 7:**
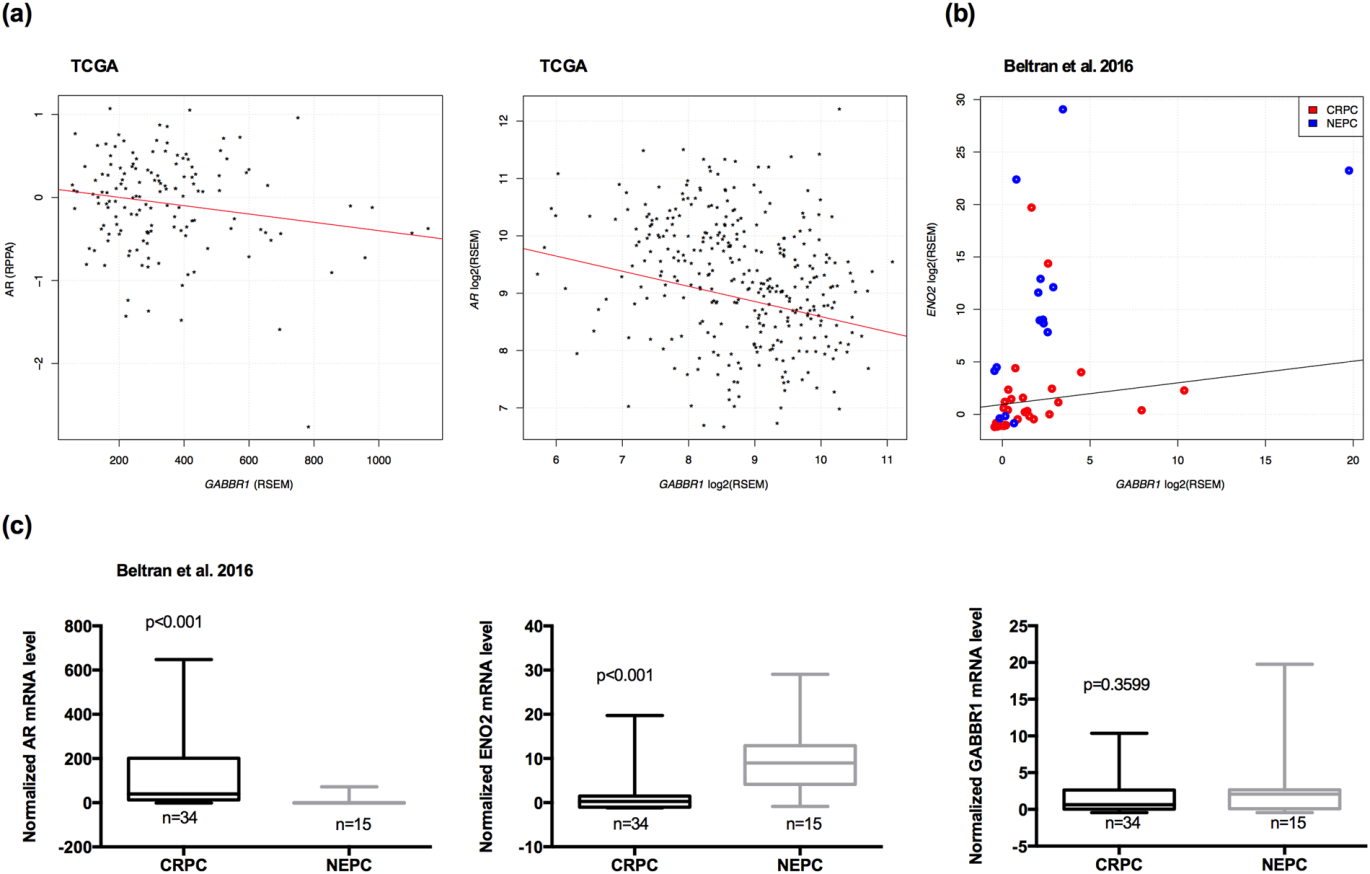
*GABBR1* is negatively correlated with AR expression and positively correlated with *ENO2* expression in NEPC. (**a**) *GABBR1* and AR expression are negatively correlated at protein level, left panel (r = −0.179; p = 0.03; n = 146) and mRNA level, right panel (r = −0.274; p = 0.003677^−4^; n = 333). Previously published prostate cancer gene and protein expression data set^40^ was acquired from cBioPortal^51,52^. *GABBR1* and AR expression values were retrieved and plotted. Previously published RNA-seq of 49 CRPC^12^ with or without neuroendocrine differentiation was obtained from cBioPortal^51,52^. (**b**) *GABBR1* and *ENO2* mRNA expression are positively correlated in NEPC group (r = 0.539; p = 0.03; n = 15) compared to CRPC group (r = 0.223; p = 0.22; n = 32). (**c**) The *AR*, *ENO2* and *GABBR1* expression values were plotted comparing NEPC to CRPC.

To evaluate further the role of *GABBR1* in PCa progression to NEPC, we analyzed *AR* mRNA levels, AR protein levels, and *GABBR1* gene expression in The Cancer Genome Atlas Research Network data set^40^. Interestingly, we observed a significant negative correlation between the expression of GABBR1 and AR, at the protein level (r = 0.179; p = 0.03; n = 146; Fig. 7a, left) and at the mRNA level (r = −0.274; p = 0.003677^−4^; n = 333; Fig. 7a, right) in primary PCa tissues. In addition, we examined a data set^12^ that included CRPC (n = 34) and NEPC (n = 15) samples to assess the regulation of *GABBR1* in NED. Importantly, we found a highly significant positive correlation between the expression of *GABBR1* and a NE marker (*ENO2*) in NEPC samples (r = 0.539; p = 0.03; n = 15; Fig. 7b) compared to CRPC samples (r = 0.223; p = 0.22; n = 32; Fig. 7b). Moreover, we observed that *AR* mRNA levels were indeed down-regulated in NEPC compared to CRPC (Fig. 7c), *ENO2* expression was upregulated in NEPC compared to CRPC (Fig. 7c), and *GABBR1* expression was similar in NEPC and CRPC (Fig. 7c). These results suggested a negative relationship between *GABBR1* and *AR* expression in patients with PCa, but a positive relationship between *GABBR1* and *ENO2* expression (a neuronal marker) in patients with NEPC.

## Discussion

This study aimed to unveil the role of GABA in the secretion of GRP, mediated by GABBR1, in NE/NE-like cells from PCa samples. We showed that both endogenous and exogenous GABA could stimulate GRP secretion in NE/NE-like cells. This stimulation promoted invasion in *AR*-deficient cells.

There is a strong relationship between alterations in AR (both mRNA and protein) levels and the progression of PCa adenocarcinoma to NEPC. Certainly, a large number of studies have shown that, under selective pressure of potent AR inhibition in PCa adenocarcinoma cells, NE-like cells transdifferentiated to form a more aggressive tumor^41–43^. Recently, Beltran *et al*., demonstrated that divergent NEPC evolution occurred in association with reduced *AR* expression, in one or more CRPC cells, through either linear or independent clonal evolution^12^. Wright *et al*. showed that AR was required to repress the NE transdifferentiation process in PCa cells *in vitro*, and they suggested that AR might actively repress an analogous NE transdifferentiation process in PCa cells *in vivo*. Following this methodology, we also confirmed that, in most cases, transdifferentiation was associated with reduced AR expression^11^. Moreover, we demonstrated that NE-like cells expressed NE markers, such as ENO2 and GRP. We also found elevated levels of GABA after knocking down *AR* in cells. Moreover, NE-like cells could secrete GABA vesicles, which enriched the conditioned medium, compared to the conditioned medium of control cells.

GABA and its receptors, GABA_A_ and GABA_B_, have been identified in NE-like cells derived from PCa samples^21^. Likewise, enriched GABA levels were found in NE cells and in primary tumors, compared to normal prostate tissues, in transgenic mice with NEPC. Also, elevated GABA levels were found in the serum of transgenic mice compared to that of normal mice^30^. Furthermore, we detected higher *GABBR1* mRNA and protein levels in NE-like cells, which implied that GABBR1 might play a role in the GABAergic pathway of NE-like cells. In addition, the GABA_B_ receptor was implicated in PCa metastasis, because it promoted MMP production^36^, metastatic spread, and treatment resistance, which was strongly associated with NEPC^44^.

Another important characteristic of tumors that display NED is the production of NE peptides, such as GRP, which play an important role in the development of castration resistance^18,37^. Studies have revealed that exogenous and endogenous GABA can stimulate the secretion of peptides such as gastrin, somatostatin and GRP in rat stomach^34^. Our results gave rise to the hypothesis that GABA and GRP secretion might be regulated by GABBR1 activity in NE-like PCa cells. Indeed, a GABA_B_ antagonist inhibited the elevation in GRP levels induced by baclofen in NE-like cells. We showed that this regulation was mediated by GABBR1, also we found that baclofen increases the mRNA and protein expression of GABBR1 in a dose-dependent manner, in NE-like cells. In future studies, it would be interesting to test whether the GABA_A_ receptor might be implicated in neuropeptide secretion, because it has been shown that GABA_A_ regulates proliferation in PCa^45^.

GRP has been identified as a potent paracrine and autocrine growth factor in PCa. Moreover, GRP promoted PCa cell migration and protease expression^46,47^. We noticed higher invasiveness rates mediated by *GRPR*. This effect was induced by GRP obtained from NE-like cells’ conditioned medium. This finding supported a correlation between NE differentiation and the increased metastatic potential of androgen-insensitive prostate cancers. Keep in mind that cancer invasion is a critical step that leads to the lethality associated with metastasis.

Another important finding from our study was the strong correlation we found between GABA and GRP in the serum of patients with PCa, particularly in serum that contained NE markers. Four out of ten patients displayed lower *AR* and higher *ENO2* gene expression levels than the levels observed in the PCa and the BPH groups, consistent with patients with NEPC in previous studies^12,48^. Moreover, the same four patients showed elevated *GABBR1* mRNA levels.

Patients with NEPC exhibited a higher Gleason score.Azuma *et al*. observed that patients with PCa metastasis had higher Gleason scores and higher prostate GABA levels compared to patients with PCa without metastasis and patients with BPH^36^. Yashi *et al*. found that patients that displayed resistance to androgen blockage showed elevated ProGRP levels in the serum. They also demonstrated that ProGRP levels in a subset of patients tended to increase when the cancer became androgen independent, but remained unchanged or decreased during the androgen-dependent stage^19^. It would be interesting to follow our patients with NEPC, who displayed elevated *GABBR1* and *ENO2* gene expression levels, to evaluate the cancer progression. This study was the first to find a direct correlation between GABA and GRP levels, particularly in the NEPC group. However, a larger number of samples would be necessary to provide a more robust statistical analysis.

We found a novel negative association between *GABBR1* and *AR* expression in PCa. Functionally, we showed that *AR* was downregulated and *GABBR1* was upregulated in NE-like cells of PCa samples derived from patients that expressed serum NE markers. Furthermore, we confirmed that these genes were negatively correlated in a PCa data set from primary tumors. As mentioned above, the presence of NED implicated reduced AR activity and/or expression. Multiple studies have suggested that genes involved in NEPC progression were accompanied, or controlled, by the loss of AR expression and/or activity^49^. Consistent with our finding of this negative correlation, we observed a high positive correlation between *GABBR1* and *ENO2* expression in NEPC samples. *GABBR1* was expressed in both CRPC and NEPC, although the expression was greater in NEPC. This result might be explained by the fact that, among the samples included in the NEPC group, some had low AR-signaling and a low NEPC classification score^12^.

Overall, our results confirmed a negative correlation between *GABBR1* expression and AR (protein and mRNA) expression. It is well known that, once CRPC progresses to NEPC, PCa cells lose the AR, which makes them resistant to any drug that targets the AR^35^. GABBR1 may represent a new therapeutic strategy for treating late-stage castrate-resistant tumors that have lost the AR and have acquired a NE phenotype. Based on our results, we propose that *GABBR1* is expressed when cells have a well-defined NE phenotype, which includes the presence of NE markers and low *AR* expression.

In conclusion, our results suggested that GABA, through GABBR1 activation in NE-like cells, induced GRP secretion, which in turn, increased cell invasion. The exact molecular mechanism through which the GABA/GABBR1 axis is involved in GRP secretion remains an important field of study and requires further investigation. However, we believe the results of this study will contribute to a better understanding of NEPC tumors.

## Methods

### Cells culture and treatment

The human prostate adenocarcinoma cell lines LNCaP and PC3 were obtained from the American Type Culture Collection (ATCC) used with passage number between 10 and 12. LNCaP was cultured in RPMI-1640 medium (Sigma-Aldrich), and PC3 was grown in DMEM medium (Corning). Both media contained 10% Fetal Bovine Serum (FBS) (Corning). Cells were maintained at 37 °C under 5% CO_2_. Baclofen at 1, 10 and 100 µM for 12 h (Sigma-Aldrich) was used to activate GABA_B_ receptor, and CGP 35348 (Sigma-Aldrich) for antagonizing GABA_B_ receptor. Cancer cells were pretreated with receptor antagonist for 2 h at concentrations of 10, 50 and 100 µM, to inhibit GRP secretion, and then incubated with GABA (100 µM) for 12 h.

### Small interfering RNA (siRNA) transfection

LNCaP cells (2 × 10^5^) were grown in phenol-red free RPMI medium (Sigma-Aldrich). Then, cells were transfected with Silencer select (Life Technologies) siRNA targeting *AR* (siRNA ID: s1538) and Negative control (siRNA ID: AM4615) using DharmaFECT 2 transfection reagent (Dharmacon) as described by manufacturer’s instructions. siRNAs final concentration was 12.5 nM per experiment. Cells were harvested every 24 h for 96 h.

### Patient cohort

A total of 10 male Mexican patients, being treated at the Hospital General Dr. Manuel Gea Gonzalez (Mexico City, Mexico) between July 2012 and February 2013, were enrolled in the present study. Written informed consent was obtained from all patients. The protocol of the present study was approved by the ethics committee of Hospital General Dr. Manuel Gea Gonzales (No.CIEI/144/12), project registration number (28-53-2012). All experiments and methods were performed according to these relevant guidelines and regulations. All patients presented palpable nodules or induration of the prostate [suspicious digital rectal examination (DRE)] and blood levels of PSA > 2.5 ng/ml. Subsequently, an experienced pathologist assessed the cores to determine whether the biopsy specimens were malignant. For those samples pathologically diagnosed as PCa, Gleason scoring was performed by careful examination of the microscopic pattern of the cancer foci. The control group comprised 6 patients with Benign Prostatic Hyperplasia (BPH), whose biopsy specimens contained benign glands and normal prostate cells. Blood and urine samples were obtained. Patient’s clinical and pathological data were collected (Table 1).

**Table 1:** Clinical features.

Pt.	Age	Gleason score	PSA (ng/ml)	Surgery	Hormone therapy
**Benign prostate hyperplasia**					
1	62	−	3.3	Prostatectomy	−
2	75	−	5.65	Prostatectomy	−
3	68	−	6.5	Prostatectomy	−
4	72	−	5.53	Prostatectomy	−
5	65	−	16.2	Prostatectomy	−
**Prostate cancer**					
A	65	3 + 3	4.87	Prostatectomy	−
B	65	4 + 4	12	TURP	−
C	64	4 + 4	4.5	TURP	−
D	83	5 + 4	31	TURP	+
E	80	3 + 3	8.4	TURP	+
F	74	3 + 3	16.0	TURP	−
G*◆	64	4 + 4	147	−	+
H*	76	4 + 3	160	−	+
I*	74	3 + 3	32	TURP	+
J*	77	3 + 3	30	TURP	+

*Prostate cancer patients with NE characteristics; ^◆^Castration-resistant prostate cancer patient; PSA, prostate-specific antigen.

### Urine sample processing and RNA extraction

Urine sample processing was performed as described^39^. The collected urine with prostate fluid was mixed with 5 ml RNAlater buffer (Qiagen) as an RNA stabilizing agent. RNA extraction was performed using the RNeasy Mini kit (Qiagen) according to manufacturer’s guidelines.

### Real-time RT-PCR assay

Total RNA from cell lines was isolated using an RNeasy kit (Qiagen) according to manufacturer’s instructions. A total of 800 ng from cell lines and 250 ng from urine samples [as described previously^39^] of RNA were reverse transcribed in a thermal cycler using SuperScript II (Invitrogen) according to manufacturer’s instructions. All reagents for TaqMan PCR, including *AR (*Hs00171172_ml), *GAPDH (*Hs02758991_gl), *KLK3* (Hs01105076_m1), *PCA3* (Hs01371939_g1), *GABBR1* (Hs00559488_m1), *GRPR* (Hs01055872_m1), *ENO2* (Hs00157360_m1) were purchased from Life Technologies. Reactions were run in a 96-well plate format using a Viia 7 Real Time PCR-System (Life Technologies). The quantitative real-time PCR results were analyzed using the 2^−∆∆Ct^ method, using *GAPDH* as housekeeping gene.

### Protein extraction and Western blot analysis

Cells were lysed with RIPA buffer (Sigma-Aldrich) with added protease inhibitors (Sigma-Aldrich). Proteins were obtained by centrifugation at 12,500 rpm at 4 °C for 30 min and quantified using an EZQ Protein Quantitation Kit (Life Technologies) according to manufacturer’s instructions. Proteins (30 µg) were separated by electrophoresis on SDS-PAGE (Bio-Rad). SeeBlue Plus2 (Invirogen) was used for sizing and visualization of the gel running pattern and protein transfer. Resolved proteins were transferred on immobilon PVDF membranes (Millipore) at 20 V for 30 min. Membranes were air-dried, wet with methanol, washed with TBS-T and blocked at 4 °C overnight in 1% (w/v) non-fat skimmed milk/TBS-T. Membranes were incubated with antibodies against AR (1:500 ab108341), GABBR1 (1:500 ab75239) from ABCAM, GAPDH (1:20000 5174 s), ENO2 (1:200 9536 s) obtained from Cell Signaling and GRP (1:500 sc-271045) from Santa Cruz Biotechnology at 4 °C overnight.

Blots were then incubated with anti-rabbit secondary antibody (1:5000 ab99697) obtained from ABCAM or anti-mouse secondary antibody (1:300 7076 s) from Cell Signaling conjugated to horseradish peroxidase (HRP) at room temperature (RT) for 2 h. Chemiluminescence signals were detected using ChemiDoc XRS system (BioRad). Western immunoblots were quantified using ImageJ software.

### Immunofluorescence

LNCaP cells were transfected as mentioned above, cells were collected every 24 h for 96 h. Next, cells were fixed with 4% (v/v) paraformaldehyde, permeabilized with1% (v/v) Triton X-100. Then, cells were blocked with 1% bovine serum albumin in PBS for 2 hat RT and labeled with rabbit polyclonal anti-GABA (1:100 A2052; Sigma-Aldrich) for 1 hat RT and mouse monoclonal anti-AR (1:50 ab9474; ABCAM) overnight at 4 °C in PBS. The anti-GABA antibody was detected with an Alexa Fluor 488 donkey anti-rabbit antibody (Invitrogen), and the anti-AR antibody was detected with an Alexa Fluor 568 anti-mouse antibody (Invitrogen), incubated for 1 h. Nuclei were stained with 4,6-diamidino-2-phenylindole (DAPI) (Invitrogen). Immunohistochemistry was observed using a Confocal Microscope (Zeiss LSM 510 Axiovert 200 M Laser Scanning).

### Preparation of conditioned medium (CM)

In order to collect conditioned medium (CM), LNCaP-siAR and LNCaP-Control were incubated in phenol-red free RPMI culture medium with different concentrations of Baclofen (1, 10, 100 and 500 µM), or vehicle (sterile water) in a humidified incubator for 12 h, after 96 h of transfection. CM was collected and centrifuged at 2000 g for 10 min at 4 °C; medium was filtered through a 0.2 µm nylon filter; then, protease inhibitors (Sigma-Aldrich) were added and immediately used or frozen at −80 °C until needed.

### Enzyme-linked immunosorbent assay (ELISA)

ELISA was used to quantify GRP concentrations in conditioned medium and serum patients. Serum samples were separated using ultra centrifuge filters 3 K (Amicon). In brief, analyses were performed in 96-well polyvinyl chloride (PVC) microtiter plates (Maxisorp, Nunc). Plates were coated at 4 °C overnight with conditioned medium. Plates were washed three times (PBS-Tween solution) and then non-specific sites were blocked with bovine serum albumin at 37 °C for 2 h. After one washing step, a monoclonal antibody specific for GRP human (0.5 µg/ml; GenScript) was added to the plates at 4 °C overnight. Plates were washed and the Anti-rabbit secondary antibody (1:8000 ab99697, ABCAM) conjugated to horseradish peroxidase (HRP) was incubated at RT for 2 h. After another wash, final reaction was determined by adding 100 µl of freshly prepared peroxidase substrate solution (TMB-ELISA; Thermo Scientific). Enzyme was blocked with 100 µl H_2_SO_4_·2 N. The optical density was measured at 450 nm using an Epoch Microplate spectrophotometer. GRP concentrations were determined according to a standard curve from synthesized peptide (GRP; GeneScript), and results were given in pg/ml.

### Invasion assay

Cell invasion assay was performed in a transwell chamber (24-well, 8μm pore size; Corning), with the bottom coated with 1 mg/ml BD Matrigel Matrix (BD Biosciences). PC3 and LNCaP cells were re-suspended in serum free DMEM medium, and 200 µl of the cell suspension (6 × 10^4^) was supplemented to the upper chamber membranes. 700 µl of indicated conditioned medium was put into the lower chamber as a chemoattractant. After 6 h or 24 h of PC3 and LNCaP cells incubation at 37 °C in a 5% CO_2_ humidified atmosphere, respectively, non-invaded cells on the surface of the membrane were removed with a cotton swab. Cells that had invaded through the filter to the lower surface were fixed with 4% paraformaldehyde and stained with 0.5% crystal violet. Each experiment was performed 3 times in triplicate. The number of invading cells was counted in 4 fields within each trans-well. Determination of the number of invaded cells was performed using ImageJ software.

### Amino acid analysis

The levels of GABA in total lysate proteins and conditioned medium were measured by HPLC as described previously by Salazar *et al*.^50^. An aliquot was used for protein determination and perchloric acid (0.6% final concentration) was added to the homogenates. Protein was sedimented in a microfuge. The supernatant was neutralized with KOH and centrifuged again to sediment the precipitated potassium perchlorate. Amino acids were derivatized with O-phthalaldehyde and after 3 min 20 µl were injected into a Beckman liquid chromatograph, using a reverse phase ODS column (25 cm + 4 mm) and a linear gradient (from 25% to 75% methanol) of a methanol mixture: 0.1 mM potassium acetate buffer, pH 5.5. The Amino acids were fluorometrically detected, and the identification and quantitation of peaks were compared according to their retention times taking into account standards for amino acid mixtures and additional internal standards.

### Statistical analysis

Gene and protein expression levels from RT-qPCR, Western blot and data from invasion assay experiments were analyzed in Graph Pad 6.0 using the two-tailed unpaired Student t-test when comparing two groups and a one-way ANOVA for several groups at once. To assess the correlation between *GABBR1* expression and AR (with gene and protein expression from TCGA and from our independent patient cohort) a test for association between paired samples, using Pearson’s product moment correlation coefficient was performed. In this context, correlation of CRPC and NEPC was assessed jointly as well as independently. The R language for statistical computing (http://www.R-project.org) was used for this analysis and statistical significance was assigned at p < 0.05.

## Electronic supplementary material

Supplementary information
