# Supplementary material for: GABA promotes gastrin-releasing peptide secretion in NE/NE-like cells: Contribution to prostate cancer progression
Source: Sci Rep. 2018 Jul 6;8:10272. doi: 10.1038/s41598-018-28538-z (PMC6035255; doi:10.1038/s41598-018-28538-z)
Supplement: Supplementary file 1 — Supplementary information [file 41598_2018_28538_MOESM1_ESM.pdf]

GABA promotes gastrin-releasing peptide secretion in NE/NE-like cells: Contribution to prostate cancer progression

Susana R. Solorzano [1], Ivan Imaz-Rosshandler [2], Ignacio Camacho-Arroyo[3], Pilar Garía-Tobilla[1], Gustavo Morales-Montor [4], Patricia Salazar [5], Maria L. Arena-Ortiz[6] and Mauricio Rodríguez-Dorantes* [1]

1. Oncogenomics Laboratory, Instituto Nacional de Medicina Genómica, CDMX 14610, Mexico.
2. DAMTP, Centre for Mathematical Sciences, University of Cambridge, Cambridge CB3 OWA, UK.
3.Unidad de Investigación en Reproducción Humana, Instituto Nacional de Perinatología-Facultad de Química, Universidad Nacional Autónoma de México (UNAM), CDMX, Mexico
4. Urology Department, Hospital General Dr. Manuel Gea González, CDMX 14080, Mexico.
5.Department of Neurosciences, Instituto de Fisiología Celular, UNAM, AP 70/253, 04510/CDMX, Mexico.
6. Ecogenomics Studies Laboratory PCT, Facultad de Ciencias, UNAM, Mexico.

**Corresponding author:* Mauricio Rodriguez-Dorantes PhD, Oncogenomics Laboratory, Instituto Nacional de Medicina Genómica, 4809 Periférico Sur, CDMX 14610, Mexico, E-mail address: mrodriguez@inmegen.gob.mx.

Supplementary Figure 1. Full-length blots/gels

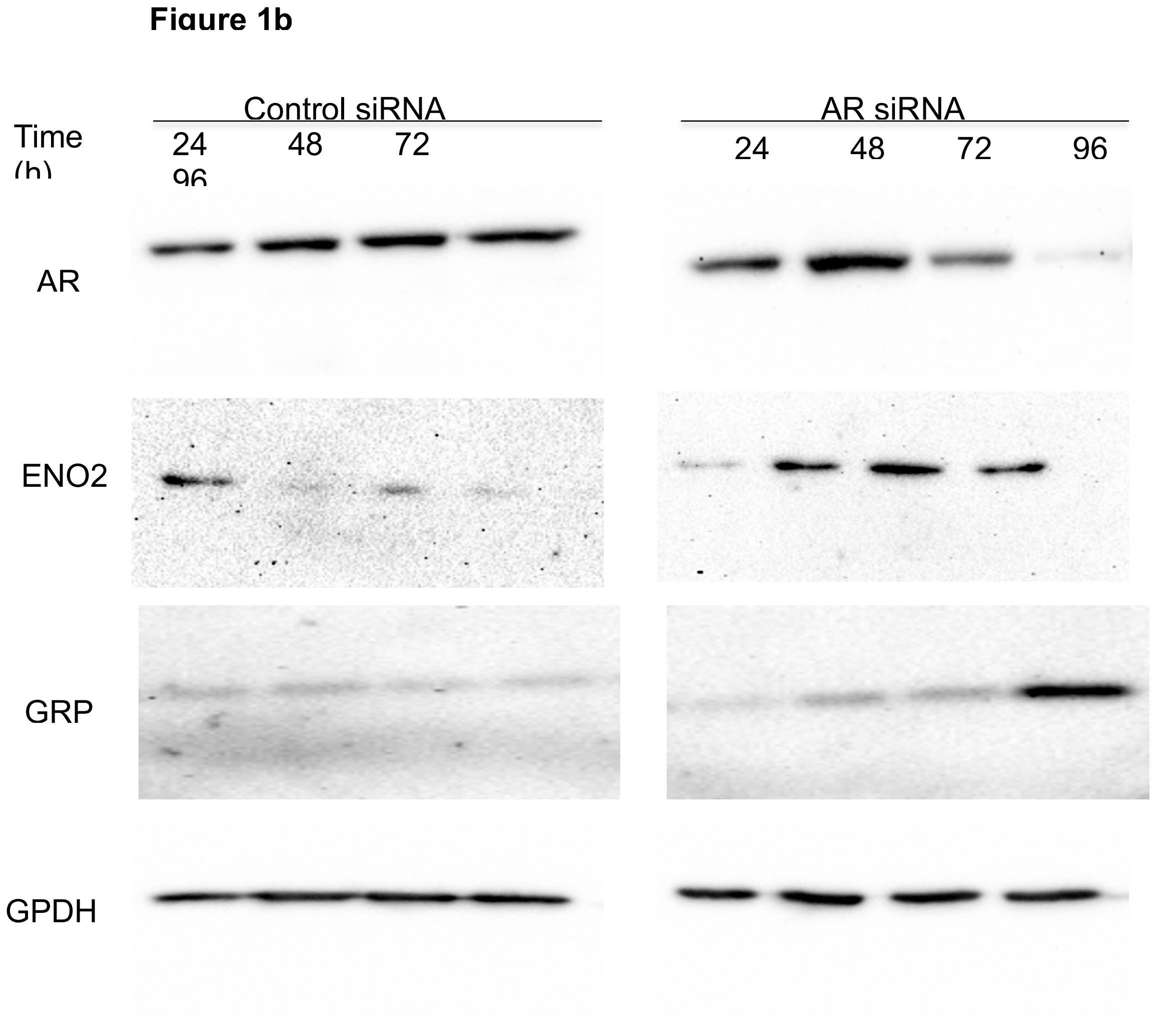

Figure 2d

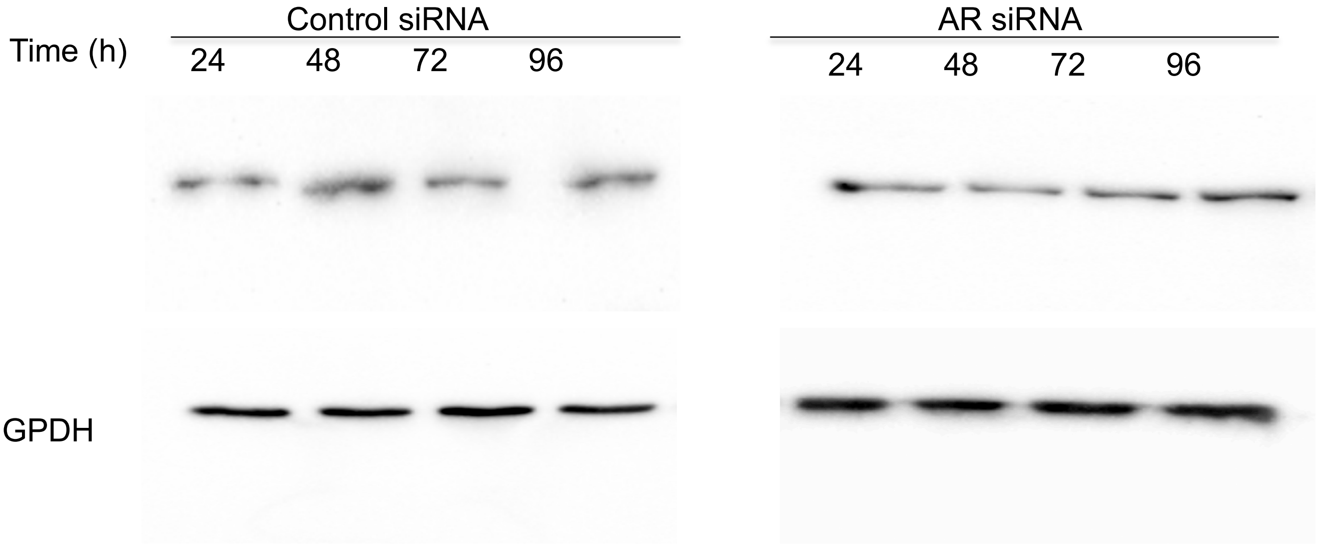

Figure 4b

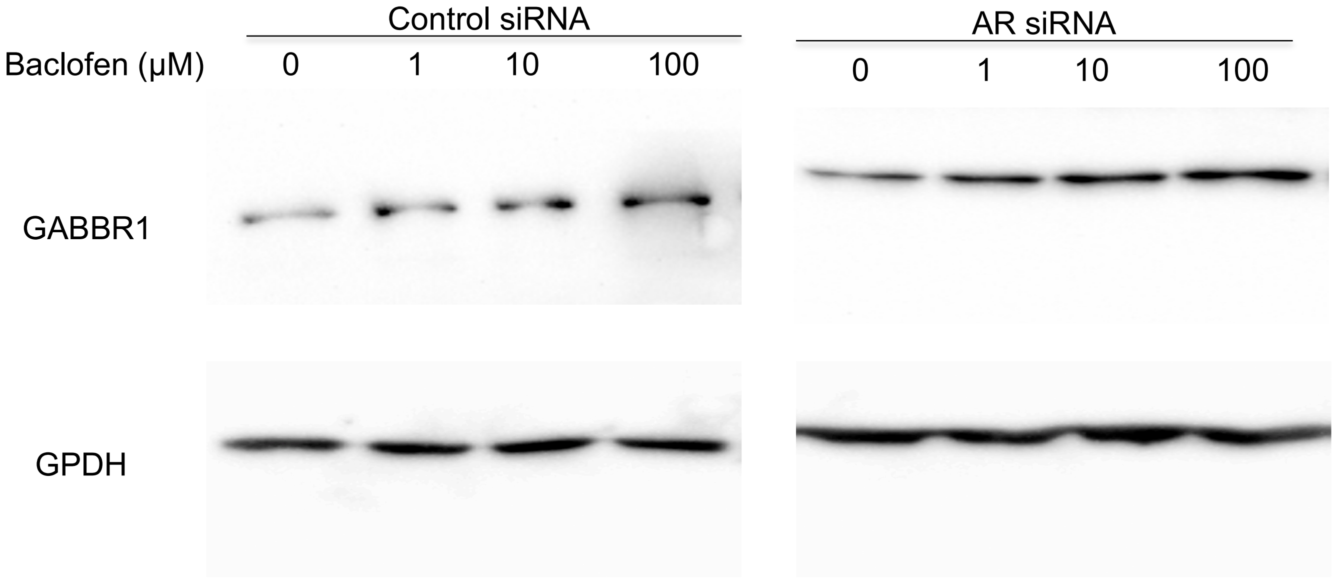

Supplementary Figure 2.

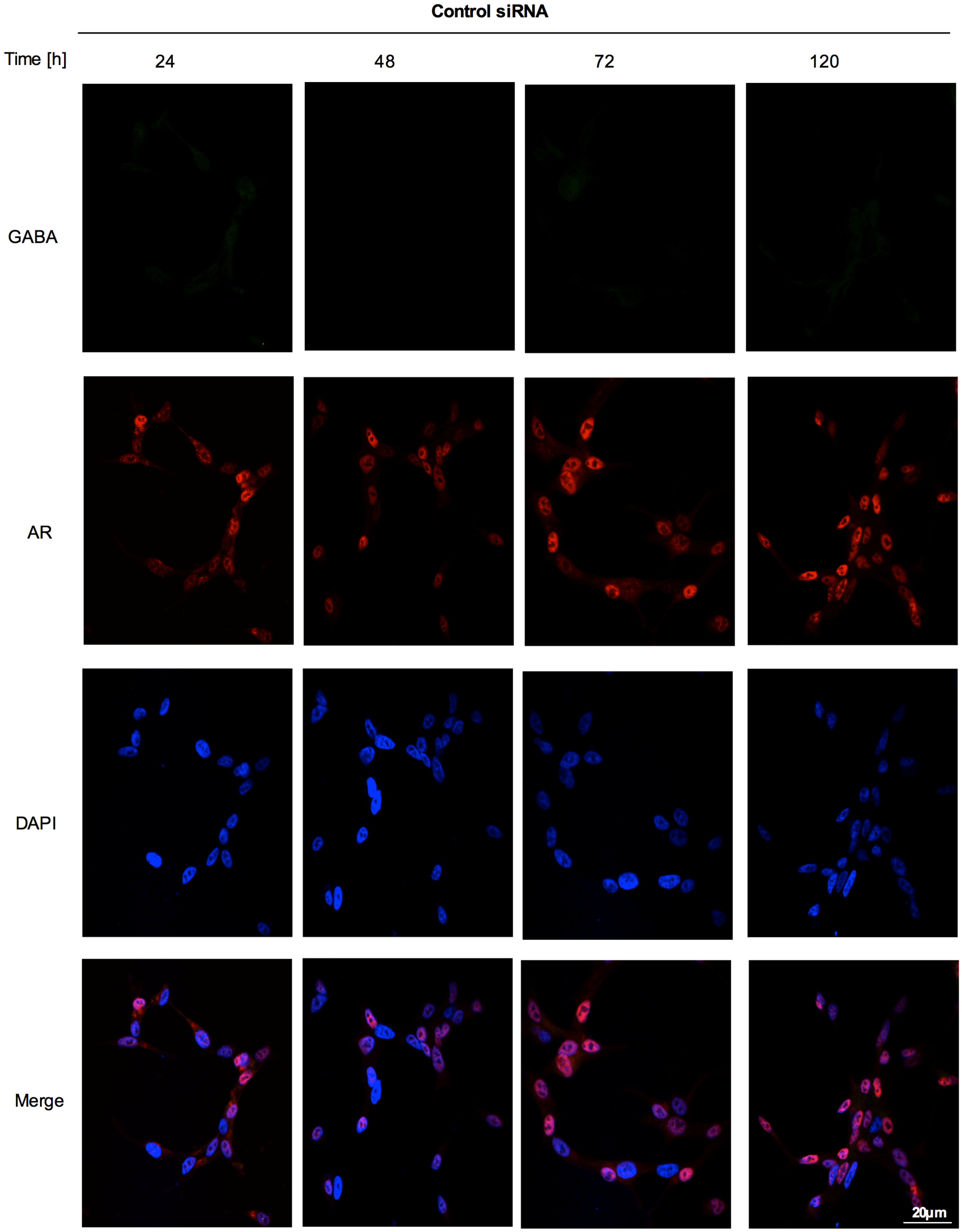

Supplementary Figure 2. Control siRNA. GABA levels are almost undetectable and AR content remains constant in control siRNA cells without morphological changes.

Supplementary Figure 3.

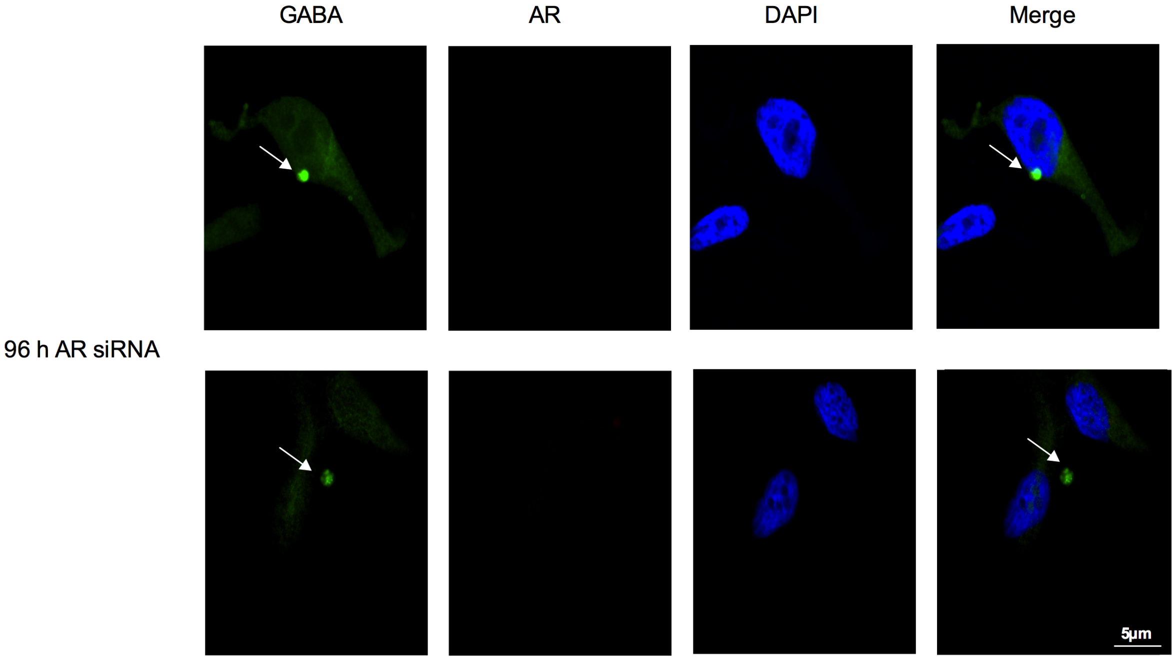

Supplementary Figure 3. Zoom of Figure 3a (scale_5μm).

Supplementary Figure 4.

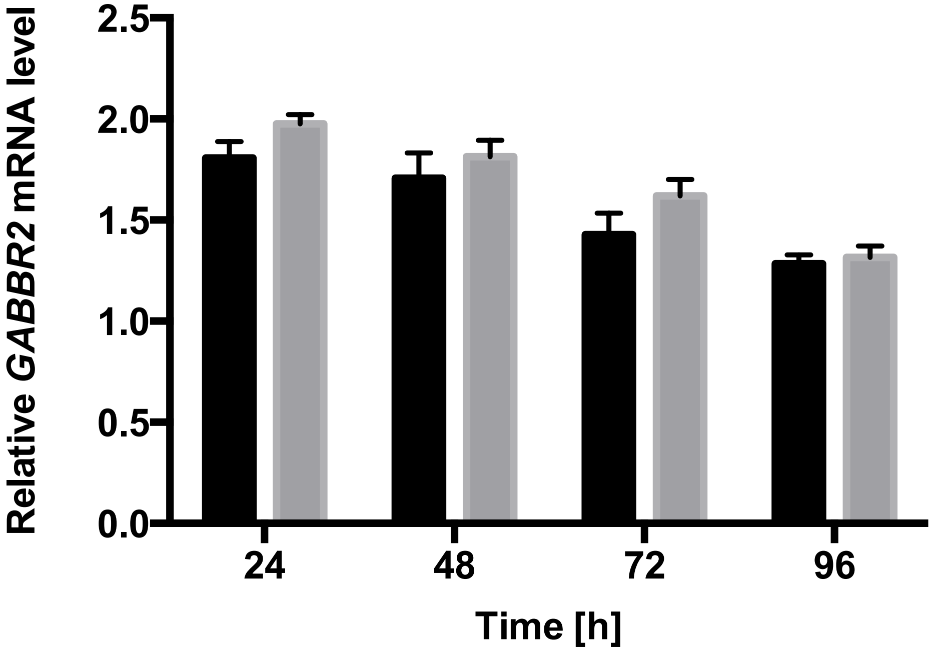

Supplementary Figure 4. GABBR2 mRNA levels. GABBR2 mRNA expression levels were analyzed by RT-qPCR in LNCaP cells transfected with AR-specific siRNA and control siRNA from 24 h to 96 h in RPMI supplemented with 10%FBS. Experiments were repeated three times. Differences among groups were compared by two-tailed unpaired Student's test. No significant differences were observed.

Supplementary Figure 5.

(a)

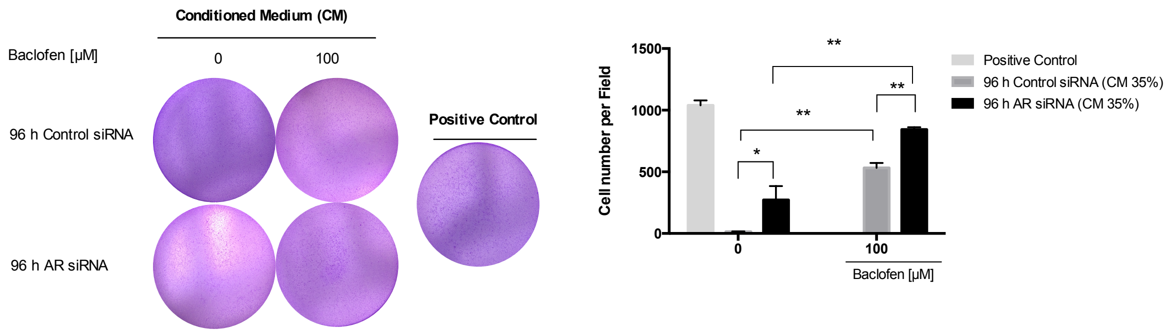

(b)

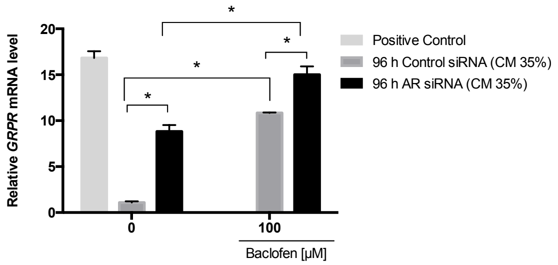

Supplementary Figure 5. The conditioned medium affects the invasive potential of NE-like cells through GRPR. a) Invasion assay was performed by using a Boyden chamber. AR knockdown LNCaP cells at 96 h ($6x10^4$) suspended in 200 μl of culture medium without FBS were placed in the upper chamber. 700 μl of conditioned medium from 96 h Control siRNA and 96 h AR siRNA treated with or without baclofen (100 μM) was added in the lower chamber for 24 h. Results are represented as mean ± SEM of three independent experiments in triplicate wells *p<0.001 and **p<0.0001. b) *GRPR* mRNA expression was analyzed by RT-qPCR. Results are presented as mean ± SEM of three independent experiments in triplicate, *p<0.0001. GRP synthetized was used as positive control; the previous concentration represents 35% of conditioned medium from 96 h AR siRNA treated with baclofen (100 μM) that was used to perform the experiments.
